# Perception of canine rabies among pupils under 15 years in Kwara State, North Central Nigeria

**DOI:** 10.1371/journal.pntd.0010614

**Published:** 2022-08-03

**Authors:** Ahmad Ibrahim Al-Mustapha, Folashade O. Bamidele, Ahmed Tijani Abubakar, Ahmed Ibrahim, Muftau Oyewo, Ibrahim Abdulrahim, Jimoh Muhammad Yakub, Idris A. Olanrewaju, Nusirat Elelu, Andy Gibson, Stella Mazeri, Muhammad Bashir Bolajoko

**Affiliations:** 1 Department of Veterinary Services, Kwara State Ministry of Agriculture and Rural Development, Ilorin, Nigeria; 2 Department of Veterinary Public Health and Preventive Medicine, Faculty of Veterinary Medicine, University of Ibadan, Ibadan, Nigeria; 3 Department of Food Hygiene and Environmental Health, Faculty of Veterinary Medicine, University of Helsinki, Helsinki, Finland; 4 African Center for Disease Control and Prevention, Addis Ababa, Ethiopia; 5 Nigeria Field Epidemiology and Laboratory Training Program, Abuja, Nigeria; 6 Department of Veterinary Public Health and Preventive Medicine, Faculty of Veterinary Medicine, University of Ilorin, Ilorin, Nigeria; 7 Mission Rabies, Cranborne, United Kingdom; 8 The Roslin Institute and The Royal (Dick) School of Veterinary Studies, Division of Genetics and Genomics, The University of Edinburgh, Easter Bush Veterinary Centre, Roslin, United Kingdom; 9 National Veterinary Research Institute, Vom, Nigeria; Universitetet i Oslo, NORWAY

## Abstract

Rabies is an endemic, highly fatal, and vaccine-preventable disease with severe socio-economic implications. Most (99%) human rabies cases are transmitted through dog bites. Children under 15 years account for 40% of all dog bite victims and 35–50% of all rabies deaths. Rabies awareness among this vulnerable group is critical to rabies prevention. However, there is a paucity of data on rabies awareness among pupils under 15. Hence, this study assessed the awareness and attitude of pupils under 15 years towards canine rabies in Kwara state in Nigeria. The study was conducted as a cross-sectional survey of 1,388 pupils across the state using a structured questionnaire that was administered as a one-on-one interview using the Open Data Kit on Android phones in December 2019. Of the 1388 pupils included in this study, only 21.7% (n = 301) of them were aware of rabies. The mean rabies score was 1.7±0.8 and only 29.2% (n = 88/301) of the pupils had adequate knowledge of canine rabies. The dog ownership rate was 18.7% (n = 259) with an average of 1.93 dogs per household. Approximately 5% (n = 66) of the pupils have been previously bitten by a dog. One-third of the dog bite victims (35%, n = 23/66) were managed and treated at home and only 12% (n = 8/66) were treated in a health facility. The result of the multivariable logistic regression showed that students aged between 13–15 years were more likely (OR: 1.93; 95% CI: 0.72–3.01; p < 0.001) to have adequate knowledge of rabies than the younger pupils. Similarly, pupils that have dogs in their households (OR: 2.09; 95%CI: 1.49–2.75; p < 0.001) and those that reside in Kwara South (OR:1.78 95% CI:1.29, 2.44; p < 0.001) were more likely to be aware and have adequate knowledge of canine rabies respectively. Finally, Pupils from non-dog-owning households were more likely (OR:2.2; 95% CI: 1.45, 4.42; p < 0.001) to have been bitten by dogs than those from dog-owning households. The awareness and attitude of pupils under 15 to canine rabies was poor. We advocate the introduction of rabies lessons into the school curriculum in Kwara State to reduce the incidence of dog bites and prevent dog-mediated human rabies.

## Author summary

Rabies is an endemic disease that kills approximately 59000 persons mostly in low- and middle-income countries. The disease is mostly transmitted through dog bites and children under 15 years account for 40% of all dog bite incidents and 35–50% of all rabies death. This study assessed the knowledge and perception of canine rabies among pupils under 15 years in Kwara State, Nigeria. Our findings showed a very low rabies awareness rate (21.7%) and equally low knowledge of rabies (29.2%) with a mean knowledge score of 1.7±0.8. The dog ownership rate was 18.7% with an average of 1.93 dogs per household. Approximately 5% of the pupils have been previously bitten by a dog. One-third of the dog bite victims were managed and treated at home and only 12% were treated in a health facility. The age of the pupil, their dog ownership status and their geopolitical location within Kwara State influenced the pupil’s knowledge of canine rabies. To control rabies, we must institute multi-sectoral collaborations of critical stakeholders and introduce rabies lessons into the school curriculum in Kwara State.

## Introduction

Rabies, the most prioritized zoonotic disease in Nigeria, poses a severe public health threat [1,2]. It is a neglected tropical disease (NTD) that is vaccine-preventable, under-reported, poorly financed, and under-diagnosed in most low-and middle-income countries including Nigeria [2,3]. Globally, rabies is responsible for an estimated 59,000 human rabies deaths and it costs over USD 8.6 billion annually [3,4]. Nigeria has an estimated ten thousand dog-bite incidents annually [5] and some 1,640 estimated human rabies cases each year [6].

The World Organization for Animal Health (WOAH) reported that children under 15 years of age account for 40% of all dog bite cases and 35–50% of all rabies deaths [2,7–9]. This could be attributed to their very curious and inquisitive nature as well as their small stature which might result to bite wounds in highly innervated areas of the body which makes it easier for the rabies virus to travel to the central nervous system [8,10].

Although the most prioritized, the public health importance of rabies has been undermined by the lack of a one-health approach to rabies surveillance, poor risk perception of rabies, as well as the lack of sufficient anti-rabies vaccines in humans (pre-exposure prophylaxis, PrEP, and Post-exposure prophylaxis, PEP) and animals (anti-rabies vaccine, ARV) [11,12]. These and several other factors have contributed to the endemicity of rabies in Kwara State and Nigeria at large. For instance, several studies have reported the poor public attitude towards animals and their welfare, poor awareness and knowledge of rabies, lack of political will, and the lack of adequate funding for rabies control [13–16]. In addition, the presence of unvaccinated free-roaming dogs (FRD) amidst human settlements is a major contributor to the high incidence and maintenance of rabies in Nigeria [17].

To reduce the incidence of dog bites and prevent dog-mediated human rabies, it is essential to assess the awareness level and further educate the general public especially children under 15 [18]. Studies have shown that “Rabies lessons” for school pupils in Northern Nigeria, the Philippines, Sri Lanka, and Malawi have increased rabies awareness among these vulnerable groups in the community and they believed that it is a cost-effective approach to rabies prevention [8,19,20]. Furthermore, the availability of effective, easy-to-use rabies information, education, and communication (IEC) tools should make the implementation of rabies lessons in Nigeria hitch-free.

The Kwara Rabies Rapid Alert System (KRRAS) is the first one-health integrated rabies surveillance project that was designed to improve the reporting of dog-bite cases and enhance the diagnosis of rabies across Kwara State. Previously, as part of the KRRAS’s baseline rabies surveillance, we have reported the estimated dog population, demography, and have assessed the public awareness and knowledge of canine rabies [17,21]. However, there is still a paucity of data on the awareness and attitude of pupils towards rabies. Hence, this study assessed awareness of canine rabies among the at-risk group (children under 15 years) across Kwara State.

## Materials and methods

### Ethics statement

This ethical clearance for this study was obtained from the ethical review boards of the Kwara State Ministry of Agriculture and Rural Development (reference number VKW/714/1/246), and the Ministry of Education and Human Capital Development, Ilorin—Nigeria (reference number: DE/PRIM/96/VOL.1/130). The ethical clearance obtained from the ERBs allowed us access to interview pupils during school hours within the school premises. Furthermore, individual written informed consent was obtained from the pupil’s guardian by giving each pupil a consent form which they took home a day before our visit. Hence, only pupils who returned filled consent forms were included in this study. Finally, all the pupils were told that they could decline participation and opt out at any time.

### Study area

Kwara State, with a human population of 3,599,800 [22] is located in the southern guinea savannah zone of Nigeria. The estimated population of children under 15 years in Kwara State is 1,583,912 (44% of the total population) [23]. The state has three agro-ecological and geopolitical zones (Northern, Central, and Southern) with vast agricultural land and forest reserves with varying climatic conditions. This study was conducted in schools in both urban (Kwara Central) and semi-urban/rural areas (Kwara North and Kwara South) of the state.

### Questionnaire design

The raw data for this study was obtained from our targeted audience (pupils under 15 years) using a structured pre-validated questionnaire. The questionnaire was validated by two independent academic examiners to ascertain the importance of the questions, the instruments’ face validity, and to ensure there were no technical hitches. Furthermore, we assessed the reliability of the survey instrument using the Cronbach Alpha test (with a score of 0.81) [24]. Finally, the questionnaire was pre-tested on twenty-five pupils from each of the senatorial zones before the deployment of the final version for data collection.

The questionnaire was composed of two sections. Section A obtained information on the pupil-related information which included their location, age, and gender. Section B assessed the awareness and knowledge of rabies among pupils. We applied skip logic to pupils who were not aware of rabies and the survey will automatically skip to section C which assessed the management of dog-bite incidents (S1 File). The questionnaire was originally designed in the English language but in some cases, it was administered in the local dialects (especially in Kwara North). This was because Kwara North had more rural communities and nomadic schools. Hence, their understanding of the English language was not as high as pupils in Kwara Central and Kwara South.

### Survey methodology

**Fig 1:**
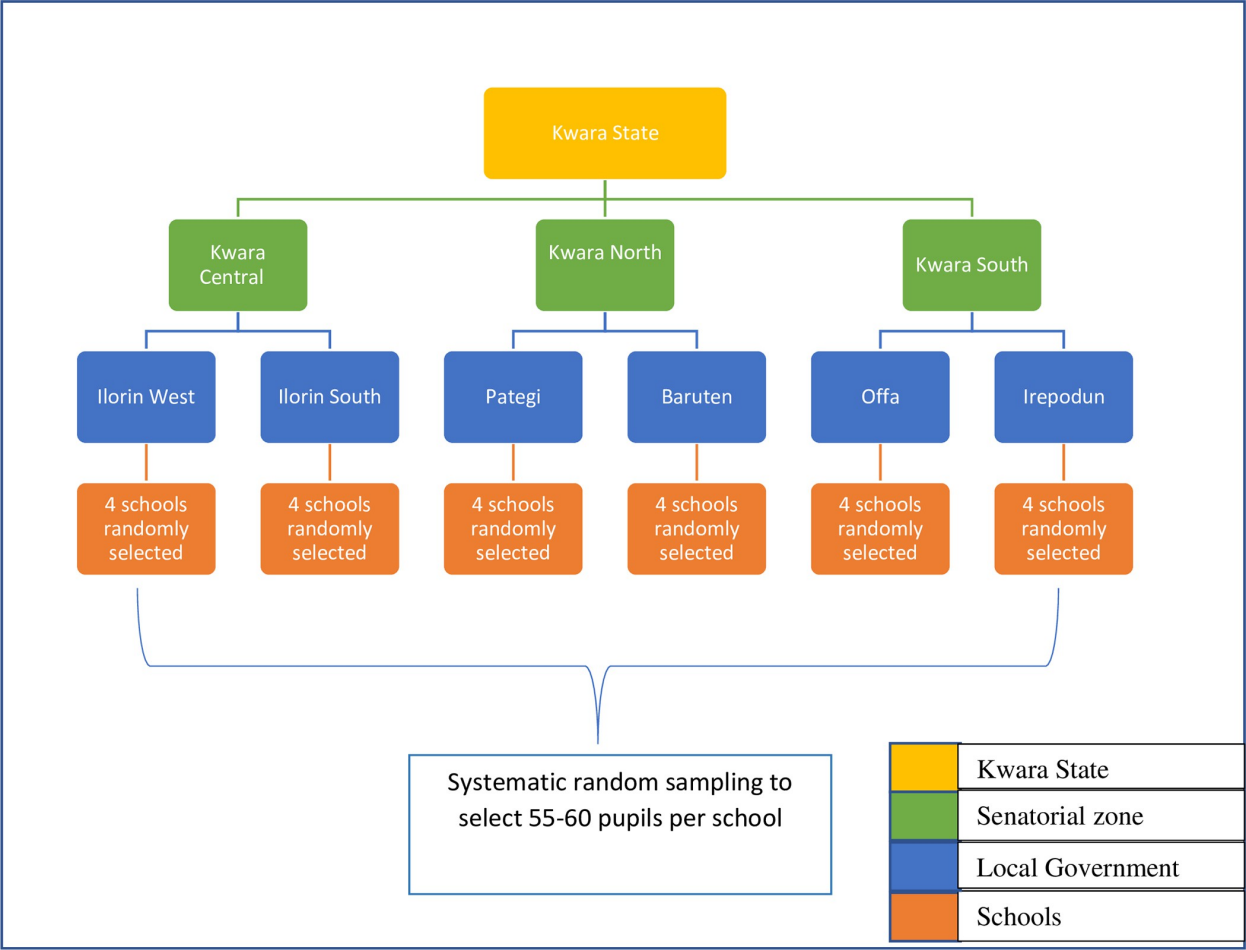
A multistage sampling of pupils across Kwara State.

The survey was conducted as a cross-sectional survey of pupils under 15 years. The survey was conducted in December 2019 in the three senatorial zones of the state. The questionnaire was administered as a one-on-one interview using the Open Data Kit (ODK) on android phones. A multi-stage sampling (Fig 1) of the schools was carried out from selected local government areas (LGAs) in the state. Four schools were selected at random from each LGA (based on the list of schools provided by the Kwara State Ministry of Education). A total of 55–60 pupils were interviewed from each of the selected schools. To prevent clustering of responses and increase the intra-cluster variability, we used the systematic random sampling technique to select each pupil (with a sampling interval of 3). The data was obtained by data clerks who were grouped into teams of 3, trained, mobilized, and purposively assigned to selected schools.

The required sample size for this study was computed using Epi-Info V.7.0 (CDC, Atlanta, USA). At a 95% confidence interval (95% CI), we hypothesized that 50% of the pupils in each of the senatorial zones would have heard of rabies. Hence, 384 pupils under 15 years were required per zone and a total of 1152 pupils for the state.

### Data analysis

The data were analyzed using Minitab v.17 (Pennsylvania, USA). Descriptive statistics of pupils’ demographic data were summarized as frequencies and proportions. To determine the pupil’s knowledge of canine rabies, a numeric scoring system was used [21]. Briefly, a correct response attracted a score of 1, while an incorrect response attracted a score of 0. Note that for questions that have multiple correct answers, pupils more mention at least 2 correct options to be allotted the 1 point. Then, we collated the sum total of each pupil’s score and graded their knowledge on a 4-item scale (cause of rabies, its symptoms, its transmission, as well as its prevention and control). Finally, we categorized each pupil as having good or poor knowledge of canine rabies using 50% of the maximum obtainable score as the cut-off. Hence, pupils who had a cumulative score of <3 (0–2 points) were graded as having poor (inadequate) knowledge of canine rabies and those who scored ≥3 (3–4 points) points were deemed to have good (adequate) knowledge of canine rabies. We used the Chi-square to test the association between pupils’ location within the state (Kwara Central, Kwara North, and Kwara South) and awareness of canine rabies, dog ownership, and prevalence of dog bites among pupils under 15 respectively. Finally, to test for association between the independent variables (e.g., age, location within the state, history of a dog bite, dog ownership, etc.) and the outcome variable (awareness of rabies among pupils under 15 years in Kwara State), we computed univariable logistic regression analysis. All variables that were significant at p < 0.05 were retained and included in the final multivariable logistic regression model.

## Results

### Demography of respondents

A total of 1,388 pupils were included in this survey. Of these, 37.9% (n = 526/1388) were from Kwara North. Most of the respondents (48%, n = 666/1388) were aged between 10–12 years and 56% (n = 777/1388) were male (Table 1).

**Table 1:** The demographic structure of pupils included in this study (n = 1388).

Variables	Frequency (%)
Senatorial zone	
Kwara North	526 (37.9)
Kwara Central	391 (28.1)
Kwara South	471 (34)
Gender	
Male	777 (56)
Female	611 (44)
Age (years)	
7–9	143 (10.3)
10–12	666 (48)
13–15	579 (41.7)

### Awareness, knowledge, and perception of canine rabies

Only 21.7% (n = 301/1388) of the pupils were aware (heard) of rabies. The source of information on canine rabies for most of the pupils was peer groups (mostly playgrounds and community meeting points (such as markets and watering points like community boreholes). The mean rabies score was 1.7±0.8 and only 29.2% (n = 88/301) of the pupils had adequate knowledge of canine rabies. Most of the students had poor knowledge of the causes of rabies, its symptoms, its mode of transmission to humans as well as its prevention and control measures (Table 2).

**Table 2:** Awareness and knowledge of canine rabies among pupils under 15 years in Kwara State, Nigeria.

Variables	Frequency (%)
1a. Are you aware (heard) of rabies?	
No	1087 (78.3)
Yes	301 (21.7)
2. What is the cause of rabies?	
Dogs	84 (27.9)
Mosquitoes	26 (8.6)
Others	17 (5.6)
I don’t know	174 (57.8)
3. What are the symptoms of rabies?	
Behavioral Changes	26 (8.6)
Gross inactivity	40 (13.3)
Inappetence	68 (22.5)
Dropped jaw	6 (1.2)
Fever	34 (11.3)
Hydrophobia	0 (0)
Paralysis	2 (0.4)
Pica	4 (0.8)
Seizures	4 (0.8)
I don’t know	211 (71)
4. How can humans contract rabies?	
Blood	12 (15)
Contact (touching or playing with animals)	19 (2.9)
Dog bites	101 (33.7)
I don’t know	169 (56.4)
5. How can rabies be prevented and controlled?	
Antibiotics	79 (26.2)
Human vaccinations	7 (2.3)
Killing stray dogs	99 (32.9)
Mass dog vaccinations	57 (18.9)
I don’t know	96 (31.9)

Of the 1388 pupils included in this study, 18.7% (n = 259) have dogs in their households. A total of 501 dogs were owned by these 259-dog-owning households. Hence, the average dog ownership was 1.93 dogs per household. Approximately 5% (n = 66/1388) of the pupils have been previously bitten by a dog. Most of the dog-bite incidents were on the lower extremities of the body (mostly on the legs), were not reported to their parents or guardians, and were associated with free-roaming dogs (stray or community-owned).

Most of the dog bite victims (pupils under 15 years) were managed as open wounds and treated at home (35%) or taken to a nearby pharmacy that prescribed pain relievers and antibiotics (21%). However, only 12% of the dog bite victims were treated in a health facility (clinic or hospital) where they could have received the rabies post-exposure prophylaxis shot. Approximately two-thirds of the dogs that bit the children were killed immediately whereas in other cases, the dogs ran away (whereabouts unknown) (Table 3).

**Table 3:** Perception of canine rabies among pupils under 15 years in Kwara state Nigeria (n = 1388).

Variables	Frequency (%)
1a. Is there a dog in your house?	
No	1129 (81.3)
Yes	259 (18.7)
1b. If yes, how many?	
1	129 (50)
2	79 (30.62)
3	18 (6.98)
4	15 (5.81)
5	7 (2.71)
6	8 (3.10
7	1(0.39)
10	1 (0.39)
2. Have you heard of rabies?	
No	1087 (78.3)
Yes	301 (21.7)
3. Have you ever been bitten by a dog?	
No	1322 (95.24)
Yes	66 (4.76)
4. Do you know anyone else (other than you) ever been bitten by a dog?	
No	1040 (75)
Yes	348 (25)
5. What did you (or your ward) do to the dog-bite wound?	
I did not inform anyone	17 (28)
They took me to the chemist (pharmacy)	14 (21)
They took me to the hospital (clinic)	8 (12)
They treated me at home	23 (35)
They said I don’t need treatment	4 (6)
6. What did you do to the dog?	
The dog ran away (whereabouts unknown)	78 (22.4)
The dog was sold	16 (4.6)
The dog was killed	211 (60.6)
Nothing happened and the dog was left alone	29 (8.4)
I don’t know	14 (4)

**Fig 2:**
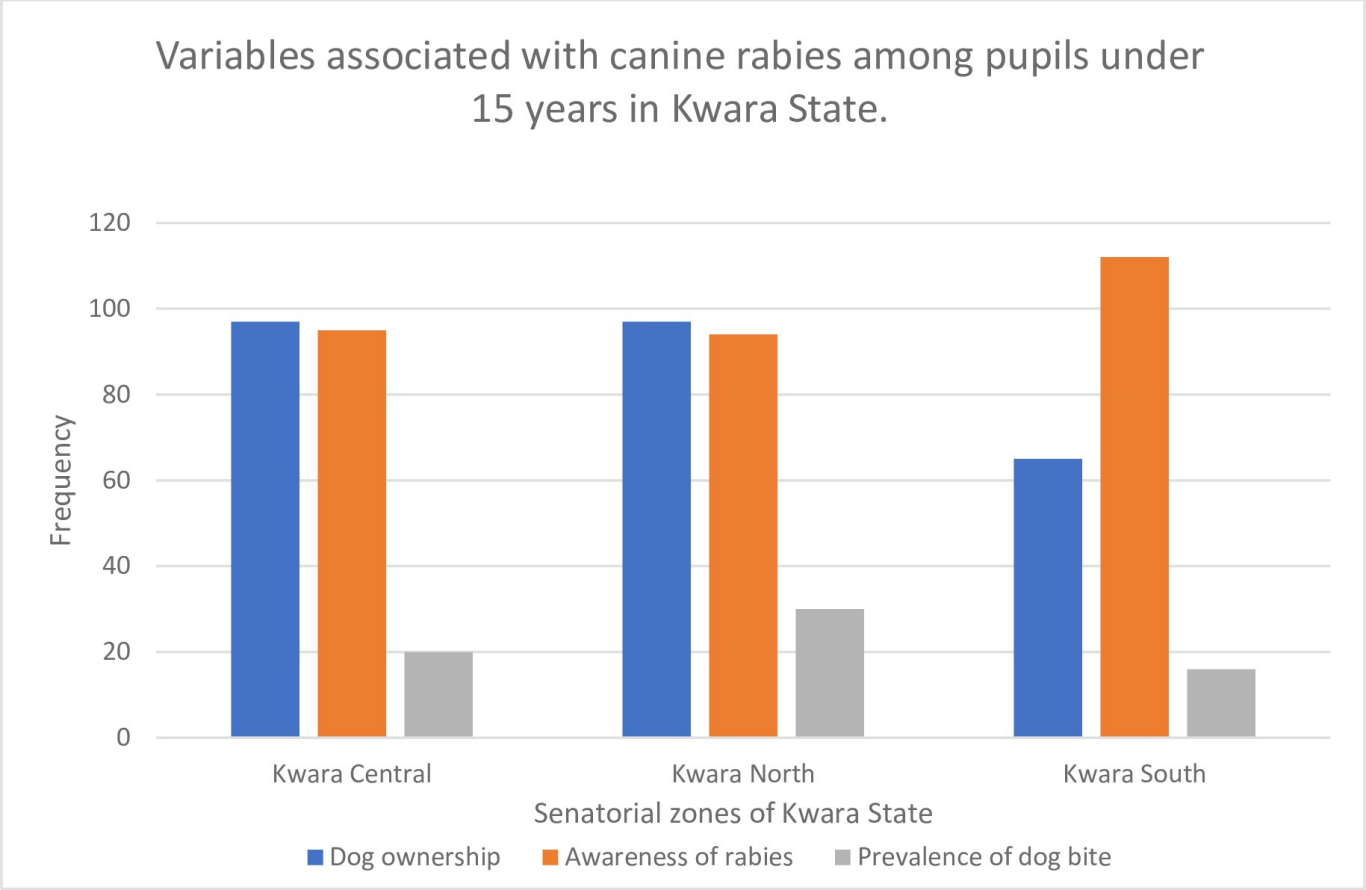
Assessment of dog ownership, awareness of rabies, and prevalence of dog bites among pupils under 15 years in Kwara State.

Across the three senatorial zones (Kwara Central, North, and South) of the state, there were no statistically significant differences between the proportion of pupils that owned dogs or the prevalence of dog bite incidents (p >0.05). However, there was a significant difference in the awareness levels of canine rabies as pupils in Kwara South were more aware of rabies than pupils in other parts of the state (Fig 2).

Of the sociodemographic variables, only the age, the dog ownership status, and the location within the state significantly influenced the awareness of canine rabies amongst pupils under 15 years in Kwara State. Our findings showed that students aged between 13–15 years were more likely (OR: 1.93; 95% CI: 0.72, 3.01; p < 0.001) to be aware of rabies than the other age groups. Similarly, pupils that have dogs in their households were more likely (OR: 2.09; 95%CI: 1.49, 2.75; p < 0.001) to be aware of rabies than pupils from households that do not have dogs. In addition, pupils in Kwara South senatorial zone were more likely (OR:1.78 95% CI:1.29, 2.44; p < 0.001) to be aware of canine rabies than their counterparts in Kwara Central and Kwara South respectively (Table 4). Pupils from non-dog-owning households were more likely to have been bitten by dogs than those from dog-owning households (OR:2.2; 95% CI: 1.45, 4.42; p < 0.001).

**Table 4:** Univariable and multivariable logistic regression analysis of demographic variables that affected the awareness of canine rabies among pupils under 15 years in Kwara state.

Outcome variable	Variable	Baseline category		OR (95% CI)	*p-value*	OR (95% CI)	*p-value*
		Univariable analysis				Multivariable analysis	
Awareness and knowledge of canine rabies	Age	7–9	10–12 13–15	1.24 (0.96, 1.62) 2.04 (1.03, 3.21)	<0.001	1.22 (0.940, 1.59) 1.93 (0.72,3.01)	<0.001
	Dog ownership	No	Yes	1.97 (1.46, 2.66)	<0.001	2.09 (1.49, 2.75)	<0.001
	Previous dog bite incident	No	Yes	0.97 (0.5305, 1.7770)	0.924	-	-
	Humans/households	1–3	4–6 >6	1.13 (0.85, 1.39) 1.02 (0.69, 1.54)	0.915	-	-
	Senatorial zone	Kwara Central	Kwara North Kwara South	1.1312 (0.82, 1.55) 1.8212 (1.33, 2.49)	< 0.001	0.96 (0.69, 1.33) 1.78 (1.29, 2.44)	< 0.001

## Discussion

Rabies is a public health threat. Its control requires multisectoral collaboration, mass dog vaccinations, intensified one-health surveillance, and mass advocacy campaigns, especially in children under 15 years. These four cardinal themes are the crux of the KRRAS [17].

This awareness rate (21.7%), as well as the knowledge of canine rabies (29.2%) among pupils under 15 years, is low. The rabies awareness rate in this study is lower than the 50.8% reported by Dzikwi et al., [8] among pupils in Zaria, North-Western Nigeria. Earlier, studies by Burdon Bailey et al [18] and Kanda et al., [20] had reported higher baseline rabies awareness rates among pupils less than 15 years in Malawi and Sri Lanka respectively. Furthermore, the awareness of canine rabies among pupils under 15 years is lower than the 38% rabies awareness rate that our previous study rate recorded in the general adult population in Kwara State [21]. The low awareness rate and knowledge of canine rabies among pupils under 15 years could be attributed to the lack of any educational program on rabies, poor mass advocacy campaigns in the state, poor risk perception of rabies among the general population, poor risk communication, and community engagement strategy in rabies control as well as the lack of an integrated rabies control program in the state [21]. Several studies have shown that public mass advocacy campaigns in schools increased rabies awareness levels in pupils [8,18,20]. This increased rabies awareness has resulted in significant improvements in rabies control programs in Sri Lanka and Ethiopia respectively [25,26].

Our findings showed that only 18.7% of all the pupils owned a dog in their household. This is consistent with our earlier finding that showed that only 20.1% of all households in Kwara state-owned a dog [17]. Furthermore, our findings showed that dog ownership was associated with significantly higher awareness and knowledge levels of canine rabies among pupils under 15 years. This could be attributed to the inquisitiveness of some dog owners to know the risks associated with keeping dogs. On the contrary, pupils from non-dog owning households were more likely to have been bitten by dogs than those from dog-owning households. The lack of training in dog handling and safety precautions around dogs could have increased the possibility of provoked dog bites among non-dog-owning households [8]. Generally, children under 15 years are at higher risk of a provoked dog bite. Generally, dog bites could be from owned dogs as well as from stray or community dogs [14,27–29] which are often very difficult to differentiate [29].

Approximately 5% of the pupils have been previously bitten by a dog. This is lower than the 13% dog bite prevalence rate that we reported amongst the general population of Kwara State [17]. This could be because in most cases, children do not report dog bite incidents to their wards (guardians or teachers in school). Hence, dog bite incidents in children under 15 years are grossly under-reported. The availability of free-roaming dogs (unconfined owned dogs, stray dogs, or community-owned) in Kwara State could have contributed to the high prevalence of dog bites [17].

The health-seeking behavior of the relatives of dog bite victims was very poor as one-third of them treated dog bite victims at home while some others took victims to a nearby pharmacy. During our previous study, we reported that dog-bite victims in Kwara State who managed the incidents at home used non-specific treatments such as wound cleaning and the use of antibiotics [17]. Previously, Atuman et al. had reported that dog-bite victims in Bauchi State, Nigeria had used traditional medication which involved roasting the liver and brain of the biting dog to be taken by the victim, the application of the dog’s hair on the bite wounds, and the use of herbs [30]. This attitude is contrary to the guidelines of the WHO which recommended that dog-bite wounds should be washed and victims should be vaccinated immediately to prevent 100% of rabies deaths [2]. Self-treatment and neglect of dog-bite wounds by some wards/guardians may have been influenced by under-reporting of dog-bite incidents by pupils, poor risk perception of rabies, and economic difficulties [8]. There is the need to continuously educate the public on the correct first aid measures upon dog bite incidents with clear messages that traditional concoctions and spiritual interventions are not the recommended first-line actions after dog-bite incidents.

Most of the pupils reported that the dog that bit them was killed. So, confirmatory diagnosis of rabies (using the brain) is usually not carried out. This act (of killing dogs) is highly discouraged and usually results in the under-reporting of rabies. We often encourage guardians of dog bite victims to quarantine the dogs or call on the local veterinary officer who should monitor the dog for 10–14 days. To protect pupils under 15 years from dog bite incidents, it is essential to control the population of stray (community) dogs [31–33].

Our findings showed that across Kwara state, there were no differences in the dog ownership status as well as the prevalence of dog bite incidents (p >0.05). This could be attributed to the availability of vast hunting areas in all parts of the state in which local dogs could be used as hunting dogs. In the same vein, the availability of community (stray) dogs in all parts of the state could be a key factor in the very similar pattern of dog-bite incidents across the state.

Our findings showed that students aged between 13–15 years and those that lived in dog-owning households were twice as likely to be aware of rabies than the other age groups. The age-dependent increase in rabies awareness could be due to the varying educational level as this age group was more likely to be in junior secondary schools while other age groups could still be in primary schools. Finally, pupils in Kwara South senatorial zone were more likely to be aware of canine rabies than their counterparts in Kwara Central and Kwara North respectively. The main strengths of this study were the wide geographical coverage and its timeliness in our effort to design a robust rabies control program in Kwara state. However, the major limitation was the possibility of the occurrence of social desirability bias.

To increase rabies awareness among pupils under 15 years, we propose the inclusion of rabies and other NTDs as part of the school curriculum. Passing key messages using “rabies lessons” as well as the distribution of information, education, and communication (IEC) materials would assist in enlightening the public on rabies. These lessons will be in-expensive, timely, and would take into consideration the socio-cultural diversity of each region of the state. Furthermore, a close collaboration between the educational authorities, human, animal, and environmental health authorities as well as non-governmental organizations is essential to successfully control rabies in Kwara State.

## Conclusion

The awareness of rabies among pupils under 15 years in Kwara State was low. The low rabies awareness rate could be attributed to the lack of mass advocacy campaigns on rabies in Kwara State. The public health threat posed by dog-bite incidents, management of dog-bite wounds, and the under-reporting of rabies would be greatly reduced if the rabies lessons were incorporated into the school curriculum.

## Supporting information

S1 File Survey material on “Awareness and knowledge of canine rabies among pupils in Kwara state”. (DOCX)

S1 Fig Geo-coordinates of schools included in this study (n = 1,388). The map was generated using QGIS v3.10.1. The shapefile was downloaded from https://www.naturalearthdata.com/downloads/.
(TIF)

## References

[1] Ihekweazu C, Michael CA, Nguku PM, Waziri NE, Habib AG, Muturi M. et al. Prioritization of zoonotic diseases of public health significance in Nigeria using the one-health approach. One Health. 2021 Dec 1;13:100257. doi: 10.1016/j.onehlt.2021.100257 34041346 PMC8144726

[2] WHO. Rabies Factsheet (2020). Available online at https://www.who.int/news-room/fact-sheets/detail/rabies.

[3] World Organization for Animal Health (WOAH). Rabies portal. http://www.oie.int/animal-health-in-the-world/rabies-portal/ Accessed 13 February 2020.

[4] Hampson K, Coudeville L, Lembo T, Sambo M, Kieffer A, Attlan M et al. Estimating the Global Burden of Endemic Canine Rabies. PLOS Neglected Tropical Diseases. 2015;9(4):e0003709. doi: 10.1371/journal.pntd.0003709 25881058 PMC4400070

[5] Nigerian Center for Disease Control (NCDC). Rabies (2018). Available online at: https://ncdc.gov.ng/diseases/factsheet/41.

[6] Alliance Rabies. Nigeria | Global Alliance for Rabies Control [Internet]. Rabiesalliance.org. 2020 [cited 8 August 2020]. Available from: https://rabiesalliance.org/country/nigeria

[7] Adedeji AO, Eyarefe OD, Okonkwo IO, Ojezele MO, Amusan TA, Abubakar MJ. Why is there still rabies in Nigeria? A review of the current and future trends in the epidemiology, prevention, treatment, control, and possible eradication of rabies. British Journal of Dairy Sciences, 2010;1(1):10–25.

[8] Dzikwi A, Ibrahim A, Umoh J. Knowledge, Attitude and Practice about Rabies among Children Receiving Formal and Informal Education in Samaru, Zaria, Nigeria. Global Journal of Health Science. 2012;4(5). doi: 10.5539/gjhs.v4n5p132 22980386 PMC4776965

[9] Sudarshan M. K., Madhusudana S. N., Mahendra B. J., Rao N. S. N., Ashwath Narayana D. H., AdbulRahman S. Assessing the burden of human rabies in India: A result of a national multi-centre epidemiological survey. *International Journal of Infectious Diseases*, 2007:11, 29–35. doi: 10.1016/j.ijid.2005.10.007 16678463

[10] Briggs D. J., & Mahendra B. J. *Public Health management of humans at risk* In: Rabies (2nd edition). Jackson AC and Wunner W.H. (eds). Academic press London, 2007:545–566.

[11] Maroof K. Burden of rabies in India: the need for a reliable reassessment. Indian Journal of Community Health. 2013; 25(4):488–91.

[12] Dodet B, Bureau ARE, Adjogoua E, Aguemon A, Amadou O, Atipo A, et al. Fighting rabies in Africa: the Africa Rabies Expert Bureau (AfroREB). Vaccine. 2008; 26(50):6295–8. doi: 10.1016/j.vaccine.2008.04.087 18617294

[13] Kia G, Huang Y, Zhou M, Zhou Z, Gnanadurai C, Leysona C et al. Molecular characterization of a rabies virus isolated from trade dogs in Plateau State, Nigeria. Sokoto Journal of Veterinary Sciences. 2018;16(2):54.

[14] Otolorin G, Umoh J, Dzikwi A. Demographic and Ecological Survey of Dog Population in Aba, Abia State, Nigeria. ISRN Veterinary Science. 2014;1–5. doi: 10.1155/2014/806849 25002978 PMC4060549

[15] Ojo D, Nwadike V, Onyedibe K, Kalu I, Ojide K. Rabies in Nigeria: A review of literature. African Journal of Clinical and Experimental Microbiology. 2016;17(2):159.

[16] Garba A, Dzikwi A, Kazeem H, Makanju O, Hambagba F, Abduazeez N et al. Dog Ecology and Management in Niger State, Nigeria: A Basic Tool for Rabies Control. Journal of Agriculture and Ecology Research International. 2017;12(1):1–9.

[17] Al-Mustapha AI., Abubakar AT., Oyewo M., Bamidele FO., Ibrahim A., Osu MS.,et al. Baseline epidemiology and associated dog ecology study towards stepwise elimination of rabies in Kwara state, Nigeria. Preventive Veterinary Medicine. 2021. doi: 10.1016/j.prevetmed.2021.105295 33611031

[18] Burdon Bailey JL, Gamble L, Gibson AD, Bronsvoort BMdC., Handel IG, Mellanby RJ, et al. (2018) A rabies lesson improves rabies knowledge amongst primary school children in Zomba, Malawi. PLoS Negl Trop Dis 12(3): e0006293. doi: 10.1371/journal.pntd.0006293 29522517 PMC5862537

[19] Lapiz SM, Miranda ME, Garcia RG et al. Implementation of an intersectoral programme to eliminate human and canine rabies: the Bohol Rabies Prevention and Elimination Project. PLoS Negl Trop Dis 2012;6:e1891. doi: 10.1371/journal.pntd.0001891 23236525 PMC3516573

[20] Kanda K, Obayashi Y, Jayasinghe A, Gunawardena GS, Delpitiya NY, Priyadarshani NG. et. al. Outcomes of a school-based intervention on rabies prevention among school children in rural Sri Lanka. International health. 2015 Sep 1;7(5):348–53. doi: 10.1093/inthealth/ihu098 25549632

[21] Al-Mustapha AI, Tijani AA, Bamidele FO, Muftau O, Ibrahim A, Abdulrahim I, et al. (2021) Awareness and knowledge of canine rabies: A state-wide cross-sectional study in Nigeria. PLoS ONE 16(3): e0247523. doi: 10.1371/journal.pone.0247523 33657138 PMC7928438

[22] National Population Commission (2020). Available online at: http://population.city/nigeria/adm/kwara/.

[23] Statista. Nigeria: age distribution of population, by gender 2021 | Statista [Internet]. Statista. 2021. Available from: https://www.statista.com/statistics/1121317/age-distribution-of-population-in-nigeria-by-gender/. Accessed 18 November 2021

[24] Tavakol M, Dennick R. Making sense of Cronbach’s alpha. International journal of medical education. 2011;2:53. doi: 10.5116/ijme.4dfb.8dfd 28029643 PMC4205511

[25] Matibag G, Kamigaki T, Kumarasiri P, Wijewardana T, Kalupahana A, Dissanayake D et al. Knowledge, attitudes, and practices survey of rabies in a community in Sri Lanka. Environmental Health and Preventive Medicine. 2007;12(2):84–89. doi: 10.1007/BF02898154 21431824 PMC2723644

[26] Jemberu W, Molla W, Almaw G, Alemu S. Incidence of Rabies in Humans and Domestic Animals and People’s Awareness in North Gondar Zone, Ethiopia. PLoS Neglected Tropical Diseases. 2013;7(5):e2216. doi: 10.1371/journal.pntd.0002216 23675547 PMC3649954

[27] Zinsstag J, Lechenne M, Laager M, Mindekem R, Naïssengar S, Oussiguéré A et al. Vaccination of dogs in an African city interrupts rabies transmission and reduces human exposure. Science Translational Medicine. 2017;9(421):eaaf6984. doi: 10.1126/scitranslmed.aaf6984 29263230

[28] Wright JC. Reported dog bites: are owned and stray dogs different?. Anthrozoös. 1990 Jun 1;4(2):113–9.

[29] Paolini A, Romagnoli S, Nardoia M, Conte A, Salini R, Podaliri. Study on the Public Perception of “Community-Owned Dogs” in the Abruzzo Region, Central Italy. Animals. 2020 Jul;10(7):1227. 10.3390/ani10071227 PMC7401527 32707663

[30] Atuman YJ, Ogunkoya AB, Adawa DA, Nok AJ, Biallah MB. Dog ecology, dog bites and rabies vaccination rates in Bauchi State, Nigeria. International Journal of Veterinary Science and Medicine. 2014 Jun 1;2(1):41–5.

[31] World Organization for Animal Health (WOAH). Stray Dog Population Control. https://www.oie.int/doc/ged/D9926.PDF Accessed 25 February 2020.

[32] Taylor L, Wallace R, Balaram D, Lindenmayer J, Eckery D, Mutonono-Watkiss B et al. The Role of Dog Population Management in Rabies Elimination—A Review of Current Approaches and Future Opportunities. Frontiers in Veterinary Science. 2017;4. 28740850 10.3389/fvets.2017.00109 PMC5502273

[33] Sabo G, Umoh JU, Sackey AKB, Ahmad A, Okolocha EC. The role of dog trade in the epidemiology of rabies: A review. *Veterinary Clinical Practice Bulletin*. (2008);1(1):63–72).

