# Supplementary material for: Perception of canine rabies among pupils under 15 years in Kwara State, North Central Nigeria
Source: PLoS Negl Trop Dis. 2022 Aug 3;16(8):e0010614. doi: 10.1371/journal.pntd.0010614 (PMC9348711; doi:10.1371/journal.pntd.0010614)
Supplement: S1 File — (DOCX) [file pntd.0010614.s001.docx]

**Awareness and knowledge of canine rabies among pupils under 15 years in Kwara State**

This study aims to assess the awareness and knowledge of canine rabies among pupils under 15 years in Kwara State. The study is essential to formulate a robust state-wide rabies prevention and control strategy to achieve global zero human rabies death by 2030. This will take 3-5 minutes. Your participation is voluntary and you can withdraw at anytime. The information will be confidential and for research purposes only. For more information, please contact [draialilori@yahoo.com](mailto:draialilori@gmail.com).

**CONSENT**

Do I have your permission to continue?

Yes (Append signature or thumbprint on consent sheet)

No

**SECTION A: PUPIL BIO-DATA**

Age (years)? ____________________________________________________

Gender: Male Female

How many people are in your house? ______________________________

Senatorial Zone: a. Kwara North

b. Kwara Central

c. Kwara South

**SECTION B: Awareness (and knowledge) of canine rabies**

1. Have you heard of rabies (locally called digbolugi, hawkan kare, gben bande)?

a. No (Skip to section C)

b. Yes (proceed with question 2)

1. What is the cause of rabies? A. Dog-bite   B. Mosquitoes C. Others D. I don’t know
2. What are the symptoms of rabies in dogs? A. Gross Inactivity B. Pica C. Inability to swallow D. Fever    E. Dropped jaw F. Behaviour changes

G. Seizures H. paralysis I. Hydrophobia J. I don’t know

1. How is rabies transmitted to humans?  A. via dog bites B. contact with dogs C. Saliva D. Mosquito bites
2. How can rabies be controlled? A. Mass dog vaccinations B. Antibiotics C. Killing of Stray dogs D. Human vaccinations E. I don’t know

**SECTION C – MANAGEMENT OF DOG-BITE INCIDENTS**

1. Do you have a dog in your house?

a. No b. Yes

1. If yes, how many? __________________________________
2. Have you ever been bitten by a dog?

a. No b. Yes

1. What did you do to the wound and what happened to the dog?
2. I did not inform anyone
3. They took me to the chemist (pharmacy)
4. They took me to the hospital (clinic)
5. They treated me at home
6. They said I don’t need treatment
7. Do you know anyone ever bitten by a dog?

a. No b. Yes

1. What happened to the person and the dog?
   1. The dog ran away (whereabouts unknown)
2. The dog was sold
3. The dog was killed
4. Nothing happened and the dog was left alone
5. I don’t know
