# Supplementary figures and images for: Perception of canine rabies among pupils under 15 years in Kwara State, North Central Nigeria
Source: PLoS Negl Trop Dis. 2022 Aug 3;16(8):e0010614. doi: 10.1371/journal.pntd.0010614 (PMC9348711; doi:10.1371/journal.pntd.0010614)

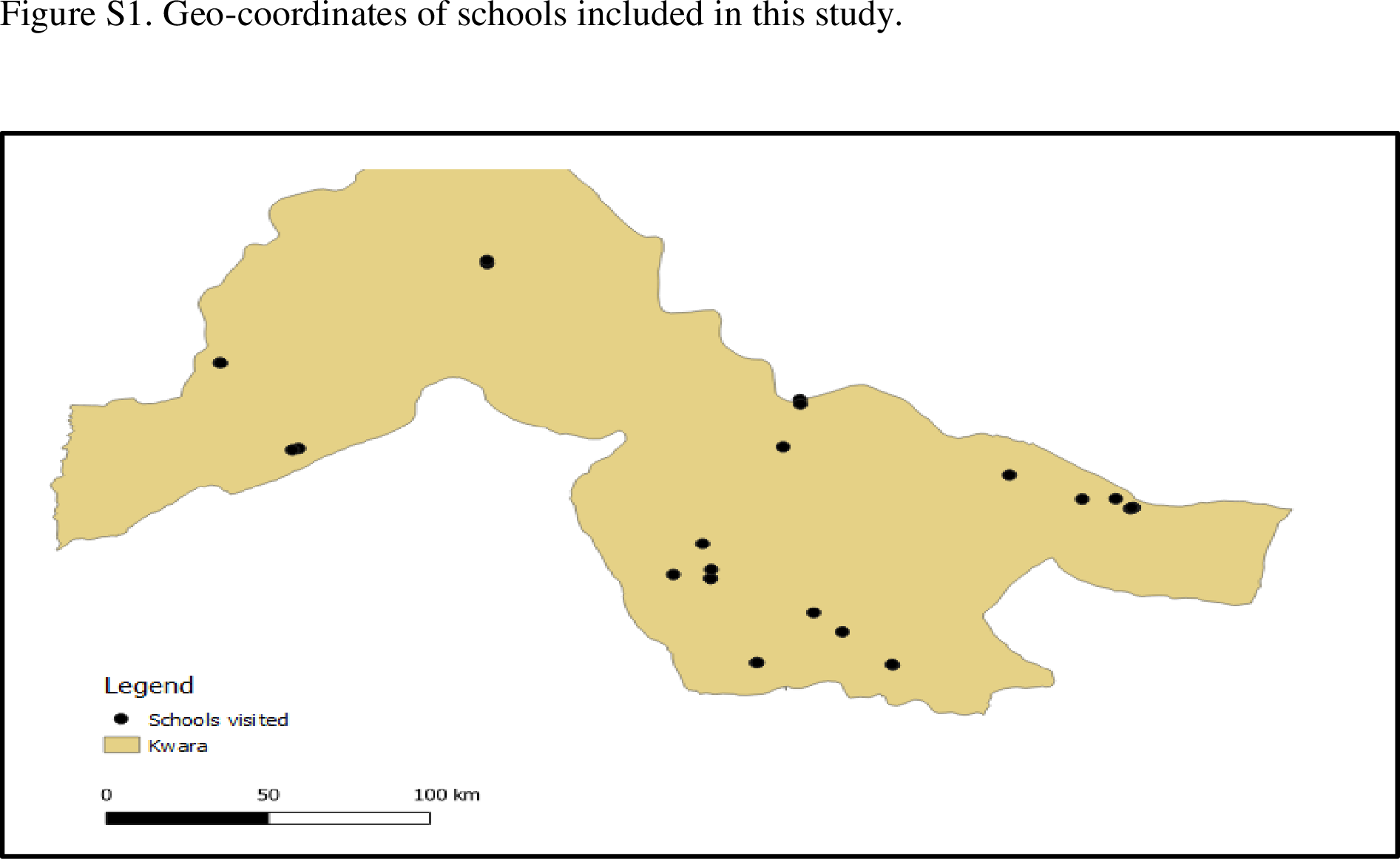

Supplement: S1 Fig — The map was generated using QGIS v3.10.1. The shapefile was downloaded from https://www.naturalearthdata.com/downloads/. (TIF) [file pntd.0010614.s002.tif]
